# Endodontic Treatment of a Mandibular Second Molar Featuring Vertucci Type V Configuration in the Distal Root: A Case Report

**DOI:** 10.7759/cureus.59905

**Published:** 2024-05-08

**Authors:** He Liu, Ya Shen

**Affiliations:** 1 Oral Biological and Medical Sciences, University of British Columbia, Vancouver, CAN

**Keywords:** cone-beam computed tomography, vertucci’s type v, ledge, root canal treatment (rct), distolingual canal, mandibular second molar

## Abstract

Successful root canal treatment relies primarily on thorough shaping, cleaning, and filling of the entire root canal system. Neglecting even a single canal can significantly raise the risk of post-treatment apical periodontitis. While the distal root of mandibular second molars typically has one canal, they can also present with anatomical variations, including the presence of a Vertucci Type V configuration. This article discusses a case in which a Vertucci Type V configuration in a mandibular second molar was effectively identified and treated.

## Introduction

Successful root canal treatment mainly depends on the adequate shaping, cleaning, and filling of all the canals in the root canal system [1-3]. Failure to address one or more canals often results in an increased incidence of post-treatment apical periodontitis [4]. Typically, the distal root of mandibular molars features one single canal, but they may also exhibit anatomical variations, such as a Vertucci Type V configuration [5,6]. 

Vertucci Type V configuration, where the main root canal divides into two independent canals in the middle to lower segment, is relatively rare, occurring in about 7-8% of mandibular first molars and about 1% of mandibular second molars [7].

Yang et al. conducted a comprehensive evaluation of the root canal morphology of mandibular second molars, analyzing morphological variations in a Chinese population using cone-beam computed tomography (CBCT) imaging. The study included a total of 1,200 bilateral mandibular second molars from 600 patients. A prevalence of 2% was reported for the Vertucci Type V configuration in the distal root [8]. Almansour et al. evaluated 304 untreated mandibular second molars in a Saudi population using CBCT imaging. However, the Vertucci Type V configuration was not observed in the distal root [9].

This case report describes a mandibular second molar with a Vertucci Type V configuration in the distal root. Utilizing CBCT and ultrasonic techniques, the Vertucci Type V configuration was successfully identified and treated. 

## Case presentation

A 22-year-old Chinese female was referred from a local dental hospital for treatment of her mandibular right second molar, tooth #47. She presented with spontaneous pain and extensive caries, leading to a diagnosis of symptomatic irreversible pulpitis. Initial root canal treatment was attempted, but the mesial canals could not be negotiated. The treating dentist temporarily filled the tooth with zinc oxide-eugenol (ZOE). At the time of consultation, tooth #47 was asymptomatic. The patient had no significant medical history and was in good health (ASA I classification), with no systemic disease or harmful oral habits. She maintained excellent oral hygiene.

**Figure 1:**
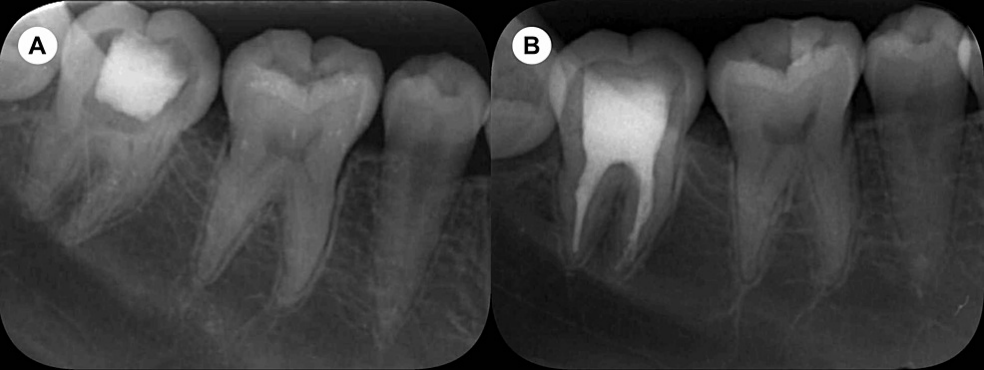
Pre-operative (A) and post-operative (B) radiographs.

**Figure 2:**
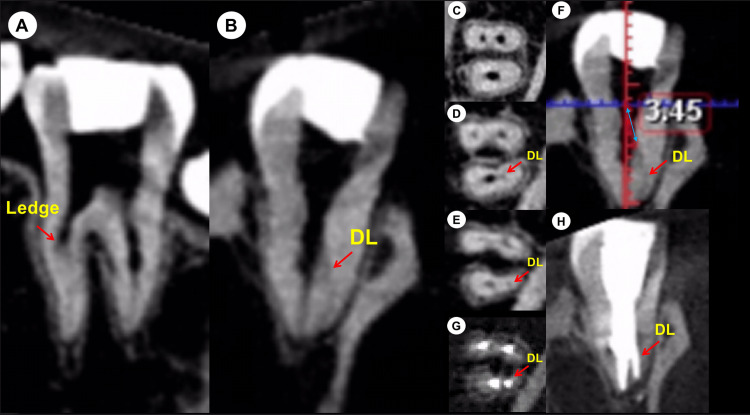
Pre-operative and post-operative CBCT images. (A) The sagittal section of the pre-operative CBCT image displayed a ledge (red arrow) in the mesial canals. (B) The coronal section of the pre-operative CBCT image revealed the emergence of a lingual branch (DL) from the midsection of the distal root canal. (C) The axial section of the pre-operative CBCT image at the coronal third of the tooth did not exhibit a DL canal. (D) The axial section of the pre-operative CBCT image at the middle third confirmed the presence of the DL canal. (E) The axial section of the pre-operative CBCT image at the apical third demonstrated the DL canal. (F) The measurement was taken from the center of the distal canal orifice to the location where the branch emerged from the midsection of the distal root canal (3.45 mm). (G) The axial section of the post-operative CBCT image at the apical third showed that the DL canal was filled. (H) The coronal section of the post-operative CBCT image confirmed that the DL canal was successfully obturated. CBCT, cone-bean computed tomography; DL, distolingual canal

Clinical examination showed normal gingival condition and no palpation tenderness at the root apex. A periapical radiograph revealed no radiopaque material in the canals and no periapical radiolucency (Figure 1A). Pre-operative CBCT scan indicated a ledge in the mesial canals (Figure 2A) and the emergence of a lingual branch (distolingual (DL) canal) from the midsection of the distal root canal (Figure 2B). The DL canal was not visible in the coronal third (Figure 2C) but was confirmed in the middle (Figure 2D) and apical thirds (Figure 2E) of the tooth. Measurements were taken from the center of the distal canal orifice to the emergence of the branch (3.45 mm) (Figure 2F). Concurrent findings led to a diagnosis of previously initiated but necrotic pulp in tooth #46. The treatment plan included root canal therapy followed by a full crown restoration to which the patient was informed and gave her consent.

After isolating the area with a rubber dam, the temporary filling was removed, and access was gained. Pre-curved K-files (#8, #10, #15) were used to navigate the mesial canals. An ultrasonic diamond tip (ET18D; Satelec Acteon Group, Merignac, France) and a stainless steel ultrasonic tip (ET21; Satelec Acteon Group) were utilized to refine the coronal third of the distal canal and locate the orifice of the DL canal. Size 8 and 10 C-Pilot files (VDW, Munich, Germany) were employed to navigate the DL canal. The working lengths of all canals were measured using an electronic apex locator (J Morita Corp, Tokyo, Japan). The canals were shaped using HyFlex CM rotary files (Coltene/Whaledent, Altstätten, Switzerland), mesial canals to #25/.04, distal canal to #40/.04, and DL canal to #25/.04. This irrigation process involved alternating between a 3% sodium hypochlorite (NaOCl) solution and a 17% ethylenediaminetetraacetic acid (EDTA) solution. Initially, the canals were soaked in the 3% NaOCl solution, followed by three 20-second ultrasonic irrigation sessions using an Irri-Safe tip (Satelec Acteon Group). Afterward, the canals were irrigated with a 17% EDTA solution, using the Irri-Safe ultrasonic tip for three more 20-second sessions. After thorough rinsing with sterile water, the canals were dried using paper points. A trial fitting of the main gutta-percha cones was conducted. Before insertion, the tips of the gutta-percha cones were dipped in AH Plus sealer (Dentsply, Maillefer, Germany). The root canal filling was completed with the continuous wave obturation technique using the Elements Obturation Unit (SybronEndo, Orange, USA). The quality of the root canal filling was radiographically assessed (Figure 1B). The axial section of the post-operative CBCT at the apical third confirmed the filling of the DL canal (Figure 2G), and the coronal section verified the successful obturation of the DL canal (Figure 2H). The cavity was restored using composite resin. The patient was advised to wait one week before proceeding with the full crown restoration.

## Discussion

The anatomical complexities of roots and root canal systems in mandibular second molars present significant challenges for endodontic procedures, particularly in locating, cleaning, shaping, disinfecting, and filling the canals [10-13]. Additionally, the prevalence of post-treatment apical periodontitis is often linked to missed canals due to incomplete eradication of the bacterial biofilm within the root canal system, which may lead to the onset or persistence of apical periodontitis [4,14]. A deep understanding of the anatomical variations of mandibular second molars is crucial for clinicians to effectively identify all roots and canals [15-17].

Research indicates that the prevalence of a distal Vertucci Type V root canal configuration in mandibular second molars varies widely, with reported instances ranging from 0% to 17% across different populations. This variation is likely due to differences in study designs and demographic factors. In a systematic review, Joshi et al. analyzed 37 articles encompassing 12,393 teeth, revealing a 77% prevalence of single canals in the distal roots, with Type I configurations most common at 85.2% and Type V configurations being exceedingly rare at 1.03% [7].

Diagnosing and visualizing such a Vertucci Type V root canal configuration remains challenging due to its rarity and detection difficulties. This configuration involves the root canal dividing into two separate canals in the middle to lower segment, creating a complex and unpredictable anatomy. This bifurcation might not be easily detected with standard X-ray imaging, complicating the treatment process. Conventional radiographs often fail to reveal dual distal canals as they may overlap buccolingually, and factors such as thick cortical bone or oblique ridges can obscure the image quality. Even with high magnification using a dental microscope, clinicians struggle to visualize the canal branching [5]. 

Limited field-of-view (FOV) CBCT imaging provides three-dimensional insights into the localization of the distal canal branching, enabling precise measurements and enhancing procedural accuracy while minimizing tooth damage [18,19]. In the reported case, the measurement was taken from the center of the distal canal orifice to the branch emergence at 3.45 mm from the midsection of the distal root canal. Periapical radiographs are typically the preferred imaging modality for immediate postoperative assessment [18]. However, in this specific case, a limited FOV CBCT was performed post-operatively to confirm the successful treatment of the distal Vertucci Type V root canal configuration. This decision was influenced by the fact that the case was referred and complex, involving a challenging Vertucci Type V configuration. The patient was informed of these challenges and requested a post-operative CBCT to ensure that the treatment was effectively administered, as periapical radiographs cannot have sufficed to confirm this.

The branches of the root canal may be very fine and tortuous, making them difficult to negotiate and thoroughly clean. Residual pulp tissue or infection can lead to treatment failure. Irrigation plays a crucial role in endodontic treatment by removing debris, dissolving residual pulp tissue, and disinfecting the root canal system to prevent reinfection. The effectiveness of this process is enhanced by the choice of irrigant, its concentration, and the irrigation technique employed [20-22].

In the reported case, an ultrasonic diamond tip (ET18D) and a stainless steel ultrasonic tip (ET21) were used to refine the coronal third of the distal canal and locate the orifice of the DL canal. Size 8 and 10 C-Pilot files were employed to negotiate the DL canal. Heat-treated, highly flexible HyFlex CM rotary files were used to shape the DL to #25/.04 using the Tactile Controlled Activation (TCA) technique developed by Chaniotis, which involves a single-stroke activation of a stationary engine-driven file, only rotating after full engagement in a patent canal to provide tactile feedback of the canal's underlying anatomy before activating the file [23]. The TCA technique, utilizing heat-treated nickel-titanium rotary instruments, enables more efficient navigation through complex canal geometries. This approach ensures thorough cleaning while reducing the likelihood of instrument separation [24,25].

## Conclusions

Effective treatment of mandibular second molars with a Vertucci Type V configuration in the distal root can be challenging. CBCT imaging plays a crucial role in identifying branching in the distal canal, allowing for precise measurements and enhancing the accuracy of the procedure.
